# Relationship between combat-related traumatic injury and its severity to predicted cardiovascular disease risk: ADVANCE cohort study

**DOI:** 10.1186/s12872-023-03605-0

**Published:** 2023-11-27

**Authors:** Christopher J. Boos, Usamah Haling, Susie Schofield, Paul Cullinan, Anthony M. J. Bull, Nicola T. Fear, Alexander N. Bennett, for the ADVANCE Study, Dan Dyball, Eleanor Miller, Stefan Sprinckmoller, Maria-Benedicta Edwards, Helen Blackman, Melanie Chesnokov, Emma Coady, Sarah Evans, Guy Fraser, Meliha Kaya-Barge, Maija Maskuniitty, David Pernet, Helen Prentice, Urszula Pucilowska, Lajli Varsani, Anna Verey, Molly Waldron, Danny Weston, Tass White, Seamus Wilson, Louise Young

**Affiliations:** 1 Academic Department of Military Rehabilitation, Defence Medical Rehabilitation Centre, Stanford Hall Estate, Near Loughborough, LE12 5QW Nottinghamshire UK; 2 The Academic Department of Military Mental Health, King’s College London, London, SE5 9RJ UK; 3 Faculty of Health & Social Sciences, Bournemouth University, Bournemouth, BH1 3LT UK; 4 Department of Cardiology, University Hospitals Dorset, Poole Hospital, Longfleet Rd, Poole, BH15 2JB Dorset UK; 5 Faculty of Medicine, National Heart and Lung Institute, Imperial College London, London, SW3 6LR UK; 6 Centre for Blast Injury Studies, Department of Bioengineering, Imperial College London, London, SW7 2AZ UK

**Keywords:** Military, QRISK3, Cardiovascular risk, Combat, Traumatic injury

## Abstract

**Background:**

This study investigated the relationship between combat-related traumatic injury (CRTI) and its severity and predicted cardiovascular disease (CVD) risk.

**Material and methods:**

This was an analysis of comparative 10-year predicted CVD risk (myocardial infarction, stroke or CVD-death) using the QRISK®3 scoring-system among adults recruited into the Armed Services Trauma Rehabilitation Outcome (ADVANCE) cohort study. Participants with CRTI were compared to uninjured servicemen frequency-matched by age, sex, rank, deployment (Afghanistan 2003–2014) and role. Injury severity was quantified using the New Injury Severity Score (NISS).

**Results:**

One thousand one hundred forty four adult combat veterans were recruited, consisting of 579 injured (161 amputees) and 565 uninjured men of similar age ethnicity and time from deployment/injury. Significant mental illness (8.5% vs 4.4%; *p* = 0.006) and erectile dysfunction (11.6% vs 5.8%; *p* < 0.001) was more common, body mass index (28.1 ± 3.9 vs 27.4 ± 3.4 kg/m^2^; *p* = 0.001) higher and systolic blood pressure variability (median [IQR]) (1.7 [1.2–3.0] vs 2.1 [1.2–3.5] mmHg; *p* = 0.008) lower among the injured versus uninjured respectively. The relative risk (RR) of predicted CVD (versus the population expected risk) was higher (RR:1.67 [IQR 1.16–2.48]) among the injured amputees versus the injured non-amputees (RR:1.60 [1.13–2.43]) and uninjured groups (RR:1.52 [1.12–2.34]; overall *p* = 0.015). After adjustment for confounders CRTI, worsening injury severity (higher NISS, blast and traumatic amputation) were independently associated with QRISK®3 scores.

**Conclusion:**

CRTI and its worsening severity were independently associated with increased predicted 10-year CVD risk.

**Supplementary Information:**

The online version contains supplementary material available at 10.1186/s12872-023-03605-0.

## Introduction

Cardiovascular disease is the leading cause of premature death and a major cause of disability in the UK [1, 2]. There is an increasing body of evidence to suggest that combat-related traumatic injury (CRTI) may be linked to increased cardiovascular disease (CVD) risk [3–5]. However, this data is based on relative low quality studies that either lacked an uninjured comparator group or when they did these were not matched by age or exposure and hence are heavily prone to bias [3, 5–7]. The recent wars in Afghanistan and Iraq have brought this issue into sharper focus. For example, in the British-led military operation in Afghanistan (2002–2014) alone 2,187 soldiers were wounded and their longer-term health consequences of these injuries remain unknown [8, 9].

The on-going ArmeD SerVices TrAuma and RehabilitatioN OutComE (ADVANCE) Study has been designed to explore the relationship between CRTI on long-term CVD and other health outcomes among this Afghanistan military cohort [10]. Recently published baseline data from the ADVANCE cohort has shown that arterial stiffness and the prevalence of metabolic syndrome was greater in veterans with CRTI compared with a frequency matched similar sized group of deployed yet uninjured veterans and were independent of age, ethnicity, time from injury/deployment and rank [4]. This risk was further enhanced by worsening injury severity.

The determination of a causal relationship between CRTI and clinical CVD will require much longer-term follow up over > 15–20 years given the current age of the ADVANCE cohort. One method that could be used to gain further early insight into the future CVD risk following CRTI is the use of composite cardiovascular risk calculators [11]. They have been shown to provide relatively accurate estimations of future CVD risk, in individuals and populations without major atherosclerotic disease. Several have been widely adopted into mainstream clinical practice to identify individuals at greater CVD risk, for targeted primary prevention strategies including early statin treatment [12]. Unfortunately, the majority of available CVD risk calculators have not been validated in younger adults and the comparative estimation of future CVD risk among a cohort of injured versus uninjured contemporary combat veterans has not been conducted [11, 12].

The QRISK®3 represents an important advance in CVD risk; it is validated across a far wider age range (25–84 years) than the majority of available CVD risk calculators with 2.67 and 7.89 million patients being included in its validation and derivation cohorts respectively [13]. Its precision is rated as excellent and particularly strong when used to estimated future CVD risk in younger adult populations [13, 14]. QRISK®3 has recently been incorporated into the National Institute for Health and Care Excellence (NICE) primary CVD prevention Guidelines with the recommendation to start statin treatment in adults without known CVD and 10-year CVD risk of > 10% [15].

In this study we sought to investigate the relationship between CRTI and future CVD risk in the ADVANCE Study Cohort. We hypothesised that CRTI would be associated with an increased 10-year predicted CVD risk compared with a matched cohort of uninjured veterans. Secondly, we hypothesised that worsening injury severity would enhance this risk.

## Methods

### Study population and design

The participants for this study consisted of the completed baseline participants included in the ADVANCE Study. ADVANCE is an on-going prospective longitudinal cohort study of adult male military personnel (aged 18–50 years at recruitment) who were deployed during Operation HERRICK (Afghanistan 2002–2014). The detailed protocol and baseline characteristics of the recruited cohort have been previously reported [16]. In brief, the final recruited cohort consists of 579 adults with CRTI (exposure) sustained during deployment to Afghanistan, who were frequency-matched to 565 uninjured men by age, service, rank, regiment, deployment period and role in-theatre. The planned duration of follow up of this cohort is expected to be ≥ 20 years. Persons with known cardiovascular disease or active infection/inflammation were excluded [16].

The baseline participant recruitment visits were conducted at the Defence National Medical Rehabilitation Centre at Headley Court, Surrey from 2016–2018 and thereafter at Stanford Hall, Nottinghamshire until completion in 2020. Prior to arrival all participants were advised to fast for ≥ 8 h and abstain from smoking for ≥ 4 h. Primary data used to quantify the predicted composite CVD risk were collected at the single baseline visit and led by a trained research nurse. socioeconomic status was estimated using military rank which was categorised into three groups based on NATO ranks as previously described: Officer rank (OF-1 to OF10), Non-commissioned Officers (other ranks [OR] OR5 to OR9) and lower/other ranks (OR1 to 4) [4].

### Calculation of predicted cardiovascular risk

Predicted CVD risk was quantified using the 21-field QRISK®3 Scoring system and calibrated for UK data [13]. This risk calculator estimates the estimated 10-year absolute CVD (confirmed coronary artery disease, myocardial infarction, stroke, transient ischaemic attack or cardiovascular-related death) risk score as well as the relative risk (compared with population expected risk). Electronic weighing scales and a stadiometer were used to record participant weight and height respectively (to determine body mass index [BMI]). An adjusted weight calculation was performed for the amputees to account for the mass of their missing limbs as previously described and validated [17]. Brachial blood pressure was measured using the Vicorder device in a temperature-controlled, noise-free environment with the arm cuff attached to the participant in a supine position with a minimum of at least three readings taken [4]. Fasted venous blood samples were processed at the validated local National Health Service Hospital laboratories. Serum creatinine was used to estimate glomerular filtration rate (eGFR) and identify underlying chronic kidney disease. Blood lipid levels were used to calculate the total cholesterol/high-density [HDL] cholesterol ratio. Glycated haemoglobin (HbA1c) and fasting blood glucose levels were used to exclude the presence of undiagnosed diabetes mellitus. Determination of erectile dysfunction was through the Arizona Sexual Experiences Scale [18]. The detailed case definitions used to calculate QRISK®3 scores are shown in Table 1.

**Table 1:** Variable definitions used to obtain QRISK®3 scores

Variable	Definition / categorical coding
Age	Documented in whole years
Sex	Only adult males included
Ethnicity	Nine categories—White or unstated, Indian, Pakistani, Bangladeshi, other Asian, Black Caribbean, black African, Chinese, other
Smoking status	Non-smoker, Ex-smoker, light smoker (< 10/day), moderate smoker (10–19/day), heavy smoker (≥ 20/day)
Diabetes	Fasting venous blood HbA1c > 48 mmol/mol or glucose of > 7.0 mmol/L or known type 1 or type 2 diabetes
1^st^ degree relative with MI or angina	1^st^ degree relative who experienced a myocardial infarction or angina under the age of 60 years
Chronic kidney disease (stage 3, 4 or 5)	Chronic kidney disease (eGFR < 60 mls/minute/BAS) or known diagnosis of and major chronic kidney disease (including nephrotic syndrome, chronic glomerulonephritis, chronic pyelonephritis, renal dialysis, and renal transplant)
Atrial fibrillation	A confirmed diagnosis of atrial fibrillation or atrial flutter including paroxysmal
Antihypertensive use	Current use of at least one antihypertensive drug
History of Migraine	A confirmed diagnosis of migraine or cluster headaches requiring specific migraine medication
Rheumatoid arthritis	A diagnosis of rheumatoid arthritis
Severe mental illness	A diagnosis of schizophrenia, psychosis, bipolar affective disease or treated depression (excluding use of amitriptyline for pain)
Systemic lupus erythematosus	A diagnosis of systemic lupus erythematosus
Atypical antipsychotics use	Use of atypical antipsychotics including amisulpride, aripiprazole, clozapine, lurasidone, olanzapine, paliperidone, quetiapine, risperidone, sertindole, or zotepine
Use of regular steroids	Current use oral or parenteral prednisolone, betamethasone, cortisone, depo-medrone, dexamethasone, deflazacort, efcortesol, hydrocortisone, methylprednisolone, or triamcinolone
Erectile dysfunction	ASEX score of 4 or more on the erectile domain or use of specific sex hormones for the treatment of erectile dysfunction in the presence of an abnormal score
Total Cholesterol/HDL ratio	Obtained from fasting venous blood
Systolic blood pressure variability	Standard deviation of the last three sequential systolic blood pressure readings
Body mass Index	Calculated as height (m)/weight (kg)^2^

*HbA1c* glycated haemoglobin, *eGFR* estimated glomerular filtration rate, *ASEX* Arizona Sexual Experiences Scale, *HDL* high-density lipoprotein, *MI* Myocardial infarction

Deployment data were obtained from the department of Defence Statistics UK. Injury details, including amputation status, were obtained through a synthesis of participant questionnaires, clinical examination and information from the Joint Theatre Trauma Registry. Injury severity was quantified using the New Injury Severity Score (NISS) which uses the sum of squares of the three most severe injuries irrespective of body region injured from the 2008 updated abbreviated Injury scale [10, 19]. Nurse-led questionnaires were used to document the participant’s background medical history and current medication use. The detailed criteria definitions for each of the included cardiovascular risk factors/identifiers to calculate QRISK®3 outputs are outlined in Table 1. The exposure date for the uninjured group (who were deployed at a similar time and in a similar role) was calculated as median time from deployment to injury for the injured group added to the deployment start date. There were 11 unavailable/missing postcodes of which 10 were in the injured groups and 45 duplicate postcodes (reflecting similar military accommodation) of which 37 were in the uninjured. Consequently, post codes were not used in the QRISK®3 calculation in order to minimise potential bias. Out of 21 × 1144 (= 24,024) data entries there was 28 missing data entries (0.1%) affecting 25 participants and these applied only to blood test results and blood pressure with no missing data for any other variables.

A time-limited QRISK®3 batch-processor was obtained from ClinRisk Ltd which was used to calculate the composite CVD risk using the ADVANCE study clinical variables obtained on the single baseline visit. After obtaining the relevant QRISK®3 scores, the data were subjected to manual quality control in which a selection of batch processed scores were randomly sampled and compared to manually calculated scores to confirm congruence. Missing data relating to the continuous variables of systolic blood pressure, serum cholesterol and high-density lipoprotein cholesterol were replaced using an automated imputation method within the QRISK3 software as previously described [13].

The primary outcome measures were the predicted 10-year QRISK®3 score (%) and the relative risk. The relative risk is calculated as the calculated absolute QRISK®3 Score divided by the expected population risk of a person of similar age, sex and ethnic group, without risk factors and a cholesterol/HDL ratio of 4.0 with a stable systolic blood pressure of 125 mmHg, and BMI of 25 kg/m^2^ (from age-matched validation cohort) [13].

#### Statistical analysis

All continuous data were inspected using frequency histograms to determine their distribution. Continuous variables were presented as mean (standard deviation) for normally distributed and median (IQR) (interquartile range) for non-normally distributed data. Two-group (injured versus uninjured) comparisons of continuous data were examined using unpaired t-tests and Mann–Whitney U tests for normally distributed and non-parametric data respectively. Similarly, one-way ANOVA and Kruskal–Wallis test were used for ≥ 3 group comparisons. Comparisons of categorical variables were undertaken using the Pearson’s χ^2^ test or the Fisher’s exact test.

General linear regression was performed using robust standard errors to investigate the influence of injury severity (dichotomised NISS above and ≤ median score), injury type (amputees versus non-amputee injured) and mechanism (blast versus other injury types eg gunshot) on the outcome of log QRISK®3 score as previously described [4]. We also undertook a sensitivity analysis to examine the relationship of NISS quartiles to QRISK®3 scores. The coefficients from the regression model were exponentiated and reported as Geometric Mean Ratios (GMR). The model was adjusted ‘a priori’ for age at injury/deployment, time from injury/deployment (hence duration of exposure) and military rank.

All statistical analyses were performed with SPSS 26.0 (SPSS, Chicago, IL, USA) and GraphPad Prism version 6.07 for Windows (GraphPad Software, San Diego, CA, USA). A significance threshold of 0.05 was used in all analyses, with *p* < 0.05 denoting statistical significance.

### Ethics and dissemination

The ADVANCE Study received approval from the Ministry of Defence Research Ethics Committee (Protocol Number: 357/PPE/12). All participants provided written consent to participate in the study. Data are available upon reasonable request. Given the sensitive nature of the participants, the data have not been widely available and would be subject to UK Ministry of Defence clearance.

## Results

We included 1144 participants which included 579 injured and 565 uninjured servicemen (Table 2). The adjusted response rates (excluding those who had died, had no known contact details or for whom no contact was attempted) were 59.6% for the injured 56.3% of the uninjured groups. The injured and uninjured participants were of similar age (overall mean age 34.14 ± 5.36 years) and ethnicity (> 90% white). The time from deployment or injury (overall 8.30 ± 2.15 years) and average systolic blood pressure (overall 128.8 ± 11.2 mmHg) were also similar (Table 2). Servicemen of junior rank were significantly younger than those of middle and senior ranks (32.3 ± 4.36, 38.2 ± 4.81 and 36.8 ± 6.03 years; *p* < 0.001) respectively. Among the injured the most common mechanism of injury was blast (75.11%). The median NISS was 12 (IQR 5–22). There were 161 limb amputees in the injured group.

**Table 2:** Comparative demographics and cardiovascular risk factors between uninjured and injured

	**Overall**	**Uninjured**	**Injured**	***P* value**
Number	1144	565	579	-
Age at assessment, years	34.1 ± 5.36	34.3 ± 5.41	34.0 ± 5.35	0.490
Age at deployment / injury, years	26.1 ± 5.23	26.5 ± 5.25	25.7 ± 5.18	0.010
Men	1144 (100%)	565 (100%)	579 (100%)	1.0
Rank: n (%)				< 0.001
-Officer rank	139 (12.2%)	79 (14.0%)	59 (10.2%)	
-Mid rank	253 (22.1%)	147 (26.0%)	106 (18.3%)	
-junior rank	752 (65.7%)	339 (60.0%)	414 (71.5%)	
Still serving: n (%)	614 (53.7%)	456 (80.7%)	158 (27.3%)	< 0.001
Mechanism of injury: n (%)				
-blast		-	435 (75.1%)	
-Other (accidents, gunshot, burns,		-	130 (24.9%)	
Time from injury/deployment, months	8.22 ± 2.15	8.21 ± 2.15	8.33 ± 2.14	0.393
Ethnicity, Caucasian, n (%)	1037	512 (90.6%)	525 (90.6%)	1.000
Smoking, n (%)				0.358
-Current smoker	245 (21.4%)	126 (22.3%)	119 (20.6%)	
-Ex-smoker	346 (30.2%)	178 (31.5%)	168 (29.0%)	
-Never smoked	553 (48.4%)	261 (46.2%)	292 (50.4%)	
Height, m	1.79 ± 0.68	1.79 ± 0.64	1.79 ± 0.71	0.245
Weight, kg	89.2 ± 13.43	87.9 ± 12.2	90.6 ± 14.4	< 0.001
Body mass index (kg/m^2^)	27.8 ± 3.68	27.4 ± 3.40	28.1 ± 3.90	0.001
Systolic blood pressure (mmHg)	128.8 ± 11.2	129.0 ± 11.2	128.7 ± 11.2	0.551
Systolic blood pressure variability (mmHg)	2.00 (1.16–3.41)	2.08 (1.16 – 3.51)	1.73 (1.16 – 3.00)	0.014
Diabetes: n (%)	8 (0.7%)	2 (0.35)	6 (1.0)	0.288
1^st^ degree relative with MI or angina < 60: n (%)	170 (14.9%)	85 (15.04)	85 (14.7)	0.868
Chronic kidney disease: n (%)	5 (0.4%)	2 (0.4%)	3 (0.5%)	1.000
Atrial fibrillation: n (%)	2 (0.2%)	1 (0.2%)	1 (0.2%)	1.000
Antihypertensive use: n (%)	13 (1.1%)	6 (1.1%)	7 (1.0%)	1.000
Migraine: n (%)	6 (5.2%)	1 (0.2)	5 (0.9%)	0.218
Rheumatoid arthritis: n (%)	0 (0.0%)	0 (0.0%)	0 (0.0%)	-
Systemic lupus erythematosus: n (%)	0 (0.0%)	0 (0.0%)	0 (0.0%)	-
Severe mental illness: n (%)	74 (6.5%)	25 (4.4%)	49 (8.5%)	0.006
Atypical antipsychotics use: n (%)	2 (0.2%)	0 (0.0%)	2 (0.5%)	0.500
Steroid tablet use: n (%)	3 (0.3%)	0 (0.0%)	3 (0.5%)	0.250
Erectile dysfunction: n (%)	100 (8.7%)	33 (5.8%)	67 (11.6%)	< 0.001
Cholesterol/HDL ratio	4.09 ± 1.38	4.03 ± 1.34	4.15 ± 1.42	0.151
QRISK3® Score, %	0.87 (0.46–0.74)	0.86 [0.44–1.67]	0.89 (0.46–1.76)	0.585
QRISK3® Relative risk ^a^	1.59 (1.14–2.39)	1.52 (1.12–2.34)	1.67 (1.16–2.48)	0.093

All of the above variables except rank, serving status and time from injury were used to calculate QRISK3 outputs

*MI* Myocardial infarction; *P* value refers to comparison between injured and uninjured groups only. Continuous data are presented as mean ± SD or median (interquartile range)

^a^ Relative to matched individuals of similar age, sex and ethnicity without CVD risk factors from UK population

The injured group had a greater proportion of servicemen of lower and middle ranks. The injured had a significantly higher body mass, body mass index (overall average 27.8 ± 3.68 kg/m2) and lower systolic blood pressure variability (Table 2). A history of severe mental health and erectile dysfunction were also more common in the injured versus uninjured groups. There were no other significant differences in QRISK®3 entry variables between the injured and uninjured groups (Table 2).

There were no statistically significant differences in QRISK®3 scores and relative CVD risk between the injured and uninjured groups (Table 2). There was no difference in the proportion of participants with QRISK®3 scores above 10% in the injured (*n* = 7, 1.2%) versus the uninjured (*n* = 8, 1.4%) groups respectively. The most severely injured (NISS > 12) and limb amputees had a greater relative CVD risk than the uninjured (Table 3). There was no significant difference in absolute QRISK®3 score or number of participants with a QRISK®3 > 10% with increasing injury severity or limb amputation (Table 3).

**Table 3:** Comparative of predicted future cardiovascular disease among uninjured versus injured by injury severity (NISS) and history of limb amputation

	**Uninjured**	**Injured**		***P* value**
		**NISS 1–12**	**NISS > 12**	
Number	565	313	266	
Age, years	34.25 ± 5.41	34.50 ± 5.50	33.46 ± 5.08	0.051
Time from deployment/ injury, years	8.22 ± 2.15	8.72 ± 2.06	7.86 ± 2.15	< 0.001
Rank: n (%)				< 0.001
-Officer rank	79 (14.2%)	31 (9.9%)	28 (10.5%)	
-Mid rank	147 (26.6%)	66 (21.1%)	40 (15.0%)	
-Junior rank	339 (59.2%)	216 (69.0%)	198 (74.4%)	
QRISK3® Score	0.86 (0.44–1.67)	0.95 (0.48–1.97)	0.86 (0.40–1.60)	0.337
QRISK3® Score > 10% n (%)	8 (1.41%)	3 (0.9%)	4 (1.5%)	0.809
QRISK3® Relative risk †	1.52 (1.12–2.34)	1.63 (1.14–2.46)	1.74 (1.19–2.54)	0.206
	**Uninjured**	**Injured non Amputees**	**Injured Amputees**	
Number	565	418	161	
Age, years	34.25 ± 5.41	34.41 ± 5.56	32.98 ± 4.60	0.012
Time from deployment/ injury, years	8.21 ± 2.15	8.62 ± 2.16	7.57 ± 1.92	< 0.001
Rank: n (%)				< 0.001
-Officer rank	79 (14.2%)	46 (11.0%)	13 (8.1%)	
-Mid rank	147 (26.6%)	86 (20.6%)	20 (12.4%)	
-Junior rank	339 (59.2%)	286 (68.4%)	128 (79.5%)	
QRISK3® Score	0.86 (0.44–1.67)	0.93 (0.58–1.81)	0.86 (0.50–1.70)	0.808
QRISK3® Score > 10%: n (%)	8 (1.41%)	5 (1.6%)	2 (0.80%)	0.641
QRISK3® Relative risk ^a^	1.52 (1.12–2.34	1.60 (1.13–2.43)	1.81 (1.24–2.74)	0.015

^a^ Relative to matched individuals of similar age, sex and ethnicity without CVD risk factors from UK population. *NISS* New injury severity score. *P* values refers to overall significance between three groups (a. injured, NISS 1–12 and > 12 or b. uninjured, injured non-amputee and injured amputees). Continuous data are presented as mean ± SD or median (interquartile range)

After adjustment for confounders CRTI, worsening injury severity (higher NISS by both median and quartiles), previous traumatic amputation and blast mechanism of injury were independently associated with QRISK®3 scores (predicted 10-year CVD risk) (Table 4 and supplementary tables 1 and 2).

**Table 4:** Results of multiple linear regression analysis for dependent variable of QRISK3® score

	**Univariable**		**Multivariable**							
			**Model 1 Injured vs Uninjured**		**Model 2 Injury severity**		**Model 3 Type of Injury/ amputees**		**Model 4 Injury mechanism**	
	**Unadjusted GMR (95% CI)**	***P* value**	**Adjusted GMR (95% CI)**	***P* value**	**Adjusted GMR (95% CI)**	***P* value**	**Adjusted GMR (95% CI)**	***P* value**	**Adjusted GMR (95% CI)**	***P* value**
Uninjured (ref)	1.00 (ref)		-		-	-	-	-	-	-
-Injured	1.03 (0.91–1.15)	0.685	1.12 (1.05–1.21)	< 0.001	-	-	-	-	-	-
Injured vs Uninjured						< 0.001				
-Uninjured (ref)	1.00 (ref)	-	-	-	-		-	-	-	-
-Injured (NISS 1–12)	1.09 (0.95–1.25)	0.231	-	-	1.12 (1.03–1.22)		-	-	-	-
-Injured (NISS > 12)	0.95 (0.820–1.10)	0.493	-	-	1.13 (1.03–1.23)		-	-	-	-
Type of Injury										
-Uninjured (ref)	1.00 (ref)	-	-	-	-	-	-		-	-
-Injured non-amputee	1.05 (0.92–1.19)	0.488	-	-	-	-	1.08 (1.00–1.16)		-	-
-Injured amputee	0.99 (0.83–1.18)	0.920	-	-	-	-	1.23 (1.10–1.37)	< 0.001	-	-
Injury mechanism										< 0.001
-Uninjured (ref)	-	-	-		-	-	-	-	-	
-Non-blast	-	-	-		-	-	-	-	1.07 (0.96–1.19)	
-Blast	-	-	-		-	-	-	-	1.14 (1.06–1.23)	
Age at injury/deployment	1.16 (1.15–1.16)	< 0.001	1.17 (1.16–1.17)	< 0.001	1.17 (1.16–1.18)	< 0.001	1.17 (1.16–1.18)	< 0.001	1.17 (1.16–1.17)	< 0.001
Time from injury, years	1.15 (1.12–1.18)	< 0.001	1.15 (1.13–1.16)	< 0.001	1.15 (1.13–1.16)	< 0.001	1.15 (1.13–1.17)	< 0.001	1.15 (1.26–1.16)	< 0.0001
NS-SEC/Rank, at sampling										< 0.001
-Officer rank (NS-SEC 1)	1.00 (ref)		-		-		-			
-Mid rank (NS-SEC 2)	1.59 (1.31–1.93)	< 0.001	1.25 (1.13–1.38)		1.25 (1.13–1.38) 1.34 (1.22–1.47)		1.25 (1.12–1.38)		1.25 (1.13–1.38)	
-Junior rank (NS-SEC 3)	0.65 (0.55–0.78)	< 0.001	1.34 (1.22–1.47)	< 0.001		< 0.001	1.33 (1.21–1.46)	< 0.001	1.34 (1.22–1.47)	

Each model has been adjusted for age at sampling age (at original injury/deployment), rank (at the time of injury/deployment) and time from injury and time from injury

*CRTI* combat-related traumatic injury, *GMR* Geometric mean ratio, *NISS* New injury severity score, *Ref* reference category

## Discussion

This is the first study to investigate the relationship between CRTI and predicted 10-year CVD risk. Overall, there was no significant difference in absolute or relative predicted CVD risk among a similar sized cohort of injured versus uninjured combat veterans frequency matched by age, sex, rank and deployment. However, the relative CVD risk, but not absolute QRISK®3 scores, were significantly higher in the amputee versus non-amputee injured and uninjured groups. After adjustment, CRTI and worsening injury severity (higher NISS and previous limb amputation) and the mechanism of blast injury were independently associated with increased QRISK®3 scores and predicted 10-year CVD risk.

There are several important factors that shaped our decision to use the QRISK®3 calculator in this study. The QRISK®3 has been validated for use in UK adults which is relevant to our UK military population [13, 14]. Secondly, QRISK®3 uses dynamically and annually updated health information to reflect changes in population characteristics and to enhance its predictive precision [12, 13]. Thirdly, it includes a broad range of ethnicities known to affect CVD risk and incorporates a far wider age range than the vast majority of other cardiovascular risk calculators [15]. Finally, QRISK®3 includes a greater number of ‘modifiable’ risk factors (eg BMI, blood pressure, smoking, mental health lipids and steroid use) than other available risk calculators [12]. Despite these enhancements a number of important modifiable risk factors, known to influence CVD risk, such as diet and exercise, are not included in QRISK®3. One reason for this might be the fact that accurate reporting of dietary and exercise data is known to be challenging, subjective and highly prone to recall bias lessening their reliability and potential use in CVD risk models [20, 21].

It is unfortunate that we were not able to include post code data in this study. This is perhaps not unsurprising given the population of combat veterans examined. Military servicemen tend to be a highly mobile population where variable postings within the UK and abroad are common. The transient colocation of addresses due to similar military accommodation was expected and borne out by the multiple duplicate post codes identified. Consequently, we could not include social deprivation information (using post codes) in our QRISK®3 data entry. However, we were able to undertake an indirect examination of the influence of socioeconomic status, using military rank. We found that lower rank status (at injury/deployment) was independently associated with increased QRISK®3. Military rank has been used as a proxy measure of socioeconomic [22]. Lower socioeconomic status is a well-established risk factor for future CVD and this concept was supported by our data [23, 24].

We did not observe a significant difference in absolute QRISK®3 scores between the amputees and severely injured on cross-sectional analysis. This may be due to the significantly lower age of these groups and might explain why a higher NISS and amputee status were independently associated with increased QRISK®3 scores after adjustment for confounding factors including sampling age. The independent association between blast injury (versus other injury mechanisms) and QRISK scores is interesting. This may relate to the fact that blast injuries were associated with the most severe injuries. The relationship between injury mechanism and health outcomes is a highly complex process as it well recognized that blast leads to a number of associated injuries including burns. There is ongoing work, with ADVANCE, investigating the relationship between detailed injury mechanisms/types and both physical activity and quality of life.

We found that that the relative CVD risk was > 1.0 for both the uninjured and injured groups in our study; in fact they were on average > 1.6 and hence well above the population expected risk [13]. The relative risk is essentially a means of interpreting our population’s risk versus on the expected UK population predicted CVD risk for persons of similar age, sex and ethnic group without known cardiovascular risk factors [13]. This could be interpreted as suggesting that military personnel are at higher CVD risk than the expected population risk. However, this cannot be confidently concluded from our data and would require a comparative examination of CVD risk among military and matched non-military adults of similar age, sex etc.

It is reassuring to note that only 15 participants (1.3%) of entire cohort has a QRISK®3 score of > 10%. It is generally recommended that a 10-year predicted CVD risk above 10% highlights high-risk individuals who should be specifically targeted for aggressive primary prevention strategies including statin treatment. Scrutiny of the CVD risk factors in our ADVANCE cohort versus the QRISK®3’s own derivation cohort used as part of its validation does, in part, explain their differences. For example the average BMI (27.8 kg/m^2^) and systolic blood pressures (128.8 ± 11.2 mmHg) were higher in our ADVANCE cohort compared with the UK population expected values [13]. The prevalence of ex-smokers (30.2% vs 15.4%), severe mental illness (6.4% vs 4.8%) and erectile dysfunction (8.8% vs 5.1%) were also higher in our ADVANCE population versus the > 3.5 million men included in the QRISK3 derivation cohort respectively [13]. It is interesting that this was observed despite the fact that the ADVANCE cohort was on average > 8 years younger (34.1 vs 42.6 years) than that of the QRISK®3 derivation cohort [13]. Whilst indirect comparisons are difficult these differences could be explained in part by more robust attainment of risk factors in the ADVANCE cohort where all participants underwent a detailed research visit to collect the QRISK3 variables. Also the specific definitions used for severe mental illness and erectile dysfunction are not identical in ADVANCE and QRISK®3 derivation cohort, with the later relying predominantly on available GP/medical records and available blood results rather than that obtained from a single study visit.

It could be argued given the high relative risk of our ADVANCE cohort that they are at genuinely higher CVD risk than that of the average UK population. This might seem surprising given the typical perception of military servicemen as fit young adult who are required to maintain relatively high standards of basic fitness. Contrary to public perception, it has been previously reported that UK servicemen may be at higher CVD risk than that of the age-matched UK population [25]. The explanation for this is complex and includes selection bias as well as cultural practices specific to military life (eg increased smoking [particularly on deployment] and alcohol consumption) and the greater representation of lower socioeconomic status linked to greater cardiovascular risk [25–27]. It is encouraging that several recent policy documents have helped to address this potential inequality [28].

The independent association between worsening injury severity and QRISK®3 scores is interesting. This complements previously published data from ADVANCE in which it was shown that CRTI and worsening injury severity was independently associated with increased arterial augmentation index (a measure of reduced arterial compliance) and metabolic syndrome [4]. Moreover, there is data suggesting that higher NISS and in particular a NISS ≥ 25 is associated with worsening all-cause mortality following traumatic injury [19]. However, this data relates to acute rather than historical traumatic injury and not selective military populations as in our study. Nevertheless we also found that the highest quartile of NISS (> 22) was independently associated with the highest QRISK®3 scores. Whether this relationship between worsening injury severity and a previous traumatic limb amputation will eventually translate into genuine adverse clinical outcomes, remains unknown and this determination as well as the potential mechanisms is the key tenet of the ADVANCE study.

One of the Achilles heals of risk prediction is the concept of prediction itself which is not an exact science and relies on complex mathematical equations to generate a 10-year CVD risk prediction using a variety of known cardiovascular risk factors [29]. It relies on accurate data entry to optimise its risk precision which has been shown to be stronger for population-related analysis as in this study than that for an individual. Hence, we cannot discount the possibility that we have underestimated or even overestimated our population’s true CVD risk. However QRISK®3 has been robustly validated and its precision has been graded as ‘excellent’ with an overall discriminatory c-statistic of > 0.84 which is highest in adults < 65 years (c-statistic ≥ 0.86) as in the ADVANCE cohort. We feel that by using contemporaneous and robustly collected data during research study visits strengthens our findings. In addition we had very little missing data [13, 14].

There are a number of additional limitations that need to be mentioned. Our sample size is relatively small and its power calculation based on ≥ 20 year long term follow up [16]. We have only included men in this study due to the very small number of female injuries during Operation HERRICK. The findings of this study focus solely on the baseline data obtained from the ADVANCE cohort, providing merely a snapshot of the cohorts CVD ‘predicted’ risk at singular point in time. Since the study is of a prospective longitudinal nature, CVD risk profiles will change over time. At present there is no justification for widespread primary CVD prevention strategies that specifically target the injured. However, the minority of participants with a QRISK®3 > 10% should be highlighted for closer primary prevention management [15]. We did not to control for multiplicity of comparisons, as our hypotheses were highly focused and all p values were reported alongside confidence intervals for interpretability. Decreasing the type I error could potentially increase the risk of a type II error. Finally, although our regression analyses were adjusted for age, time from injury/deployment, rank and ethnicity there is the possibility of unmeasured or residual confounding which may have resulted in biased estimates.

In conclusion, CRTI and its worsening severity, depicted by increasing NISS and a history of traumatic amputation, and blast mechanism of injury is independently associated with an increased 10-year predicted CVD risk score using the QRISK®3 scoring system. Long term follow up of this cohort is required to determine whether this increased estimated risk will translate into genuine adverse cardiovascular events or need for targeted primary prevention strategies for the injured.

### Supplementary Information

**Additional file 1:** **Supplement Table 1.** Influence of Injury severity (by quartiles) to Estimated Cardiovascular Risk. **Table 2.** Results of multiple linear regression analysis to examine the relationship between NISS Quartiles on QRISK3^®^ scores.

## Data Availability

Only the authorised authors (CJB, UH and SS) had access to the data of this study. Given the sensitive nature of the participants, data have not been made widely available. Requests for data will be considered on a case-by-case basis and subject to the UK Ministry of Defence clearance. More information can be found at: https://www.advancestudydmrc.org.uk/
