# Supplementary material for: Relationship between combat-related traumatic injury and its severity to predicted cardiovascular disease risk: ADVANCE cohort study
Source: BMC Cardiovasc Disord. 2023 Nov 27;23:581. doi: 10.1186/s12872-023-03605-0 (PMC10680223; doi:10.1186/s12872-023-03605-0)
Supplement: Supplementary file 1 — Additional file 1: Supplement Table 1. Influence of Injury severity (by quartiles) to Estimated Cardiovascular Risk. Table 2. Results of multiple linear regression analysis to examine the relationship between NISS Quartiles on QRISK3® scores. [file 12872_2023_3605_MOESM1_ESM.docx]

Supplement Table 1 Influence of Injury severity (by quartiles) to Estimated Cardiovascular Risk

|  | **Uninjured** | **NISS quartiles** | | | |  |
| --- | --- | --- | --- | --- | --- | --- |
|  |  | **1^st^**  **(NISS 1-5)** | **2^nd^**  **(NISS6-12)** | **3^rd^**  **(NISS 13-22)** | **4^th^**  **(NISS >22)** | **P**  **value** |
| Number | 565 | 145 | 168 | 125 | 141 |  |
| Age, years | 34.3±5.41 | 34.52±5.58 | 34.5±5.44 | 33.7±5.30 | 33.3±4.90 | 0.178 |
| Time from deployment/ injury, years | 8.21±2.15 | 8.86±1.89 | 8.61±2.19 | 8.12±2.10 | 7.63±2.19 | <0.001 |
| Rank: n (%)  -Officer rank  -Mid rank  -Junior rank | 79 (14.2%)  147 (26.6%)  339 (59.2%) | 14 (9.7%)  35 (24.1%)  96 (66.2%) | 17 (10.1%)  31 (18.5%)  120 (71.45) | 14 (11.2%)  19 (15.2%)  92 (73.6%) | 15 (10.65)  21 (14.9%)  105 (74.5%) | 0.006 |
| QRISK3^®^ Score | 0.86 (0.44-1.67) | 0.95 (0.45-1.76) | 0.95 (0,50-2.12) | 0.82 (0.36-2.12) | 0.95 (0.45-1.65) | 0.588 |
| QRISK3^®^ Score >10% n (%) | 8 (1.41%) |  |  |  |  | 0.809 |
| QRISK3^®^ Relative risk † | 1.52 (1.12-2.34) | 1.53 (1.11-2.42) | 1.69 (1.16-2.48) | 1.73 (1.11-2.30) | 1.76 (1.20-2.65) | 0.297 |
| †Relative to matched individuals of similar age, sex and ethnicity without CVD risk factors from UK population. NISS, New injury severity score. P values refer to overall significance between three groups; l NISS, New injury severity Score. P value refers to comparison across the uninjured and injured groups by quartiles . Continuous data are presented as mean ± SD or median (interquartile range) | | | | | | |

Table 2 Results of multiple linear regression analysis to examine the relationship between NISS Quartiles on QRISK3^®^ scores

|  | **Univariable** | **P Value** | **Mutivariable** | **P Value** |
| --- | --- | --- | --- | --- |
| Age at injury/deployment, years | 1.16 (1.15-1.16) |  | 1.17 (1.16-1.17) | <0.001 |
| Time from deployment/ injury, years | 1.15 (1.12-1.18) |  | 1.15 (1.13-1.17) | <0.001 |
| Injury severity by NISS quartile  -Uninjured (reference)  1^st^ NISS quartile  2^nd^ NISS Quartile  3^rd^ NISS quartile  4^th^ NISS quartile | -  1.07 (0.89-1.28)  1.12 (0.94-1.32)  0.92 (0.76-1.12)  0.98 (0.81-1.19) | 0.495  0.196  0.244  0.847 | 1.09 (0.99-1.21)  1.14 ((1.03-1.27)  1.06 (0.95-1.18)  1.19 (1.06-1.33) | <0.001 |
| Rank: n (%)  -Officer rank  -Mid rank  -Junior rank | 1.00 (ref)  1.59 (1.31-1.93)  0.65 (0.55-0.78) | <0.001  <0.001 | -  1.25 (1.13-1.17)  1.34 (1.21-1.47) | <0.001 |
| Ref, reference category; Each model has been adjusted for age at sampling age (at original injury/deployment), rank (at the time of injury/deployment) and time from injury. | | | | |
